# Economic Evaluation of a Novel Lung Cancer Diagnostic in a Population of Patients with a Positive Low-Dose Computed Tomography Result

**DOI:** 10.36469/001c.121512

**Published:** 2024-09-17

**Authors:** Michael J. Morris, Sheila A. Habib, Maggie L. Do Valle, John E. Schneider

**Affiliations:** 1 Pulmonary/Critical Care Service, Department of Medicine Brooke Army Medical Center, JBSA Fort Sam Houston, Texas, USA; 2 Division of Pulmonary Diseases and Critical Care Medicine Audie L. Murphy Memorial VA Hospital, UT Health San Antonio, UT Health Long School of Medicine, San Antonio, Texas, USA; 3 Avalon Health Economics, Coral Gables, Florida, USA

**Keywords:** cost-offset analysis, cost saving, economic evaluation, lung cancer diagnostic, lung cancer, indeterminate pulmonary nodules

## Abstract

**Background:** Early detection of lung cancer is crucial for improving patient outcomes. Although advances in diagnostic technologies have significantly enhanced the ability to identify lung cancer in earlier stages, there are still limitations. The alarming rate of false positives has resulted in unnecessary utilization of medical resources and increased risk of adverse events from invasive procedures. Consequently, there is a critical need for advanced diagnostics after an initial low-dose computed tomography (LDCT) scan. **Objectives:** This study evaluated the potential cost savings for US payers of CyPath® Lung, a novel diagnostic tool utilizing flow cytometry and machine learning for the early detection of lung cancer, in patients with positive LDCT scans with indeterminate pulmonary nodules (IPNs) ranging from 6 to 29 mm. **Methods:** A cost offset model was developed to evaluate the net expected savings associated with the use of CyPath® Lung relative to the current standard of care for individuals whose IPNs range from 6 to 29 mm. Perspectives from both Medicare and private payers in a US setting are included, with a 1-year time horizon. Cost calculations included procedure expenses, complication costs, and diagnostic assessment costs per patient. Primary outcomes of this analysis include cost savings per cohort and cost savings per patient. **Results:** Our analysis showed positive cost savings from a private payer’s perspective, with expected savings of 895 202 311percohortand6460 per patient, across all patients. Scenario analysis resulted in cost savings of 890 829 889percohort,and6429 per patient. Similarly, savings of 378 689 020percohortor2733 per patient were yielded for Medicare payers, across all patients. In addition, scenario analysis accounting for false negative patients from a Medicare payer perspective yielded savings of 376 902 203percohortand2720 per patient. **Discussion:** The results suggest substantial cost savings, primarily due to reductions in follow-up diagnostic assessments and procedures, and highlight the importance of accurate diagnostic tools in reducing unnecessary healthcare expenditures. **Conclusion:** CyPath® Lung utilization yields savings for private and Medicare payers relative to the current standard of care in a US setting for individuals with 6 to 20 mm IPNs.

## INTRODUCTION

Lung cancer remains the leading cause of cancer-related deaths in the United States (US). In 2023, approximately 128 000 deaths were expected as a result of lung cancer, and approximately 240 000 new cases were expected to be diagnosed.^1^ Low-dose computed tomography (LDCT) is the recommended method of screening, and studies show a significant relative reduction of 16% in lung cancer mortality, especially among high-risk patients.^2^ This includes individuals who have a 20 pack-year or more history of smoking, are between 50 to 80 years old, and have not abstained from smoking for more than 15 years.^3^ Although recognized for its precision in identifying small nodules, some clinicians have expressed concerns regarding the relatively high rates of false positives and overdiagnosis by LDCT-mediated lung cancer screening.^4^

Pulmonary nodules are identified in approximately 1.6 million people per year in the United States.^5^ Clinical management is dependent on nodule characteristics such as size, shape, location, and probability of malignancy.^6^ Risk assessment is heavily reliant on the expert clinician’s evaluation, in combination with current nodule management guidelines.^7^ Based on the 2021 CHEST Guideline and Panel Report (CHEST Guidelines),^6^ patients with indeterminate pulmonary nodules (IPN) ranging between 6 to 8 mm, regardless of risk level, will receive a follow-up LDCT between 6 to 12 months. Nodules greater than 8 mm that are assessed as being at low to moderate risk of malignancy are recommended to be followed up with functional imaging with positron emission tomography (PET) and computed tomography (CT) surveillance. However, if clinical pretest probability and imaging findings are discordant, clinicians may opt for a nonsurgical biopsy. Furthermore, in cases where IPNs are greater than 8 mm and assessed as being at a high risk probability of malignancy (>65%), the CHEST Guidelines recommend surgical diagnosis in lieu of functional imaging.^8^ The evaluation of IPNs includes use of computed tomographic imaging, PET imaging, tissue biopsies, or a combination of the above procedures depending on the size of the nodule, the probability of lung cancer, patient preference, and overall health of the patient.^9,10^

Despite the impact of increased lung cancer screening on reducing mortality, it has concurrently resulted in increased false-positive rates in patients. In an analysis done on factors influencing false-positive rates, using Lung Imaging Reporting and Data System (Lung-RADS) categories, the false-positive rate was estimated to be approximately 13.5%.^11^ The consequence of increased false-positive results includes not only the psychological and economic burden from unnecessary follow-up care but also costs associated with complications from utilization of avoidable invasive procedures, such as pneumothorax and hemorrhaging. In a study of approximately 344 000 patients, the estimated complication rate was 23.8%, with mean incremental costs varying from $6320 to $56 845 for minor and major complications.^12^

The objective of this study is to evaluate savings associated with utilization of a novel lung cancer diagnostic, CyPath® Lung (bioAffinity Technologies, Inc.). CyPath® Lung is a diagnostic tool that uses flow cytometry and automated data analysis developed with machine learning for the early detection of lung cancer.^13^ Patients collect their sputum sample at home over 3 consecutive days and send the sample overnight to the laboratory. CyPath® Lung uses the patient’s sputum to look for 3 markers indicative of the presence of lung cancer. Overall, CyPath® Lung performed with a sensitivity of 82% and specificity of 88% compared with an LDCT specificity of 73.4%.^13–15^ CyPath® Lung performed equally well in detecting cancer in individuals with small nodules 20 mm or less, showing 92% sensitivity and 87% specificity, with an area under the curve of 92% and 99% negative predictive value. Novel diagnostics such as CyPath® Lung aid physicians in establishing an optimal care plan in a timely manner and can help to avoid potentially invasive or unnecessary tests and procedures.

## METHODS

**Figure 1.**
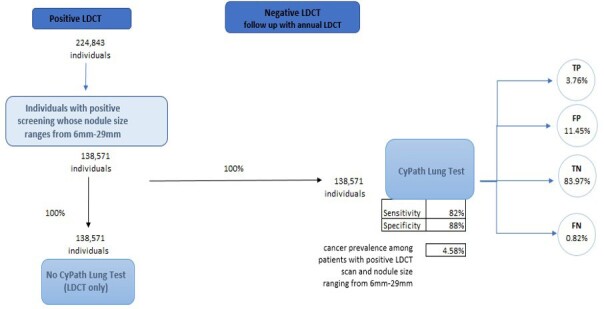
Conceptual Model Abbreviations: FN, false negative; FP, false positive; LDCT, low-dose computed tomography; TN, true negative; TP, true positive.

The population of interest for this cost analysis includes individuals with a positive LDCT whose pulmonary nodules range from 6 to 29 mm. This range was chosen as a result of its clinical significance. This range of nodule size represents stages of lung cancer where early detection and treatment have the potential to significantly improve outcomes and survival rates, especially in patients with smaller nodules. We structured the economic evaluation as a cost offset analysis, which is often used as an economic evaluation method to assess financial impacts of new healthcare interventions by directly comparing cost of intervention against the cost savings generated.^16^ The analysis calculated total savings by taking into account both the direct costs of implementing CyPath® Lung and the resulting cost reductions from reducing healthcare expenditures. A conceptual model was then created from the US payer perspective while assuming a 1-year time horizon (**Figure 1**). Prevalence data from the American Lung Association was used to derive an initial cohort reflective of the US population (n = 823 600 individuals). This value represents the 5.8% of eligible Americans who were screened in 2021, out of the estimated total of 14.2 million Americans who meet the guidelines for screening.^17^ We used this initial value of 823 600 individuals with a positive LDCT scan rate of 27.3% to calculate the total number of expected individuals with a positive LDCT scan (n= 224 843) (**Figure 1**).^18^

To estimate the number of individuals who had both a positive LDCT scan and a pulmonary nodule that ranged from 6 to 29 mm, we utilized National Lung Screening Trial data provided by Gierada et al.^19^ In that study, 61.63% of the 26 309 initial LDCT screening scans showed a nodule size that ranged from 6 to 29 mm, with a cancer prevalence of 4.58%. Two pathways are presented in our model: (1) current standard of care (SOC), which does not include the CyPath® Lung test after the initial positive LDCT; and (2) CyPath® Lung test used after initial positive LDCT. The model simulates the net effects of 100% of the cohort moving through each arm of the model. The sensitivity and the specificity of CyPath® Lung, in addition to cancer prevalence in the population, are then used to calculate the number of individuals who are expected to be categorized as true positive (TP), false positive (FP), true negative (TN), and false negative (FN).

The model focused on the likely utilization of five procedures throughout the follow-up period as a component of the care pathway: (1) CT; (2) PET; (3) CT-guided biopsy; (4) bronchoscopy; and (5) surgical biopsy. To determine the likelihood of receiving each given procedure during our time horizon, we used data that indicated a patient’s overall probability of undergoing a biopsy within 1 year and multiplied that value by the probability of a patient receiving any of the 5 procedures mentioned above (**Table 1**). Data were abstracted from a study by Zhang et al, where the medical management of patients with suspicious pulmonary nodules (SPN) and patients with a confirmed lung cancer diagnosis (LCDx) were evaluated.^20^ Considering the prevalence rate of cancer modeled in this analysis, we found it important to model expected follow-up expenses for both patients with SPN and patients who are expected to receive a primary lung cancer diagnosis. This was an important distinction in the analysis given that these 2 groups have different probabilities of undergoing each procedure as well as varying expected costs during follow-up. Other parameters included the average number of procedures undergone by each patient, sensitivity and specificity of both LDCT and CyPath® Lung, and the expected complication rates per procedure including hemorrhaging and incidence of pneumothorax. The reported sensitivity and specificity for CyPath® Lung is 82% and 88%, respectively.^13^

**Table 1.** Probability of Receiving a Given Procedure Each Year Among Individuals With SPN and Confirmed Diagnosis of Lung Cancer

**Procedures and Tests**	**SPN, %**	**LCDx, %**
Probability of receiving a biopsy each year	6.15	82.89
Average procedure (count) per patient	1.43	1.18
Probability of receiving procedure by biopsy type^a^		
CT-guided biopsy	19.32	42.75
Bronchoscopy	69.14	48.33
Surgical biopsy	11.55	8.92
Probability of receiving procedure		
Chest imaging	61.80	72.53
PET/CT	0.62	1.34
CT-guided biopsy^b^	1.19	35.43
Bronchoscopy^b^	4.25	40.06
Surgical biopsy^b^	0.71	7.39

Source: Zhang et al.^20^

^a^Based on patients who received a biopsy.

^b^Probability of biopsy procedures are multiplied by the probability of receiving a biopsy in a given year. Abbreviations: CT, computed tomography; PET, positron emission tomography.

To assess complication rates associated with each procedure, we conducted a comprehensive literature search of complication rates associated with each procedure in real-world clinical studies. Based on the studies reviewed, we calculated an unweighted average complication rate for each procedure (**Table 2**). Rates varied from 19% to 39% for CT-guided biopsy, 2.3% to 14.3% for bronchoscopy, and 11.3% to 11.6% for surgical biopsies.^12,21–26^

**Table 2.** Procedure-Related Complication Rates

**Complication Rates**	**Reference**	**Rate, %**
Computed tomography–guided biopsy	22	25.8
	23	27.2
	24	39.0
	25	19.0
Average		**28.0**
Bronchoscopy	26	2.3
	27	3.1
	25	14.3
Average		**6.6**
Surgical	12	11.3
	25	11.6
Average		**11.5**

The model perspectives were Medicare and private payers in the US, with the model designed to produce separate results for each perspective. Base prices for procedures were based on 2023 Medicare national payment data by current procedural terminology (CPT) codes obtained from public data sources. We then used a 2.64 multiplier to estimate costs for private/commercial payers.^27^ Furthermore, incremental outpatient costs associated with complications were collected from an economic study conducted by Vachani et al.^21^ We focused on the 3 most common complications for these procedures, which included incidence of pneumothorax, pneumothorax requiring a tube, and hemorrhage. We used an unweighted average cost of complications for this analysis. Finally, we calculated a total average expected diagnostic assessment cost per patient, for both individuals with an SPN and individuals with an LCDx. This final value was calculated by multiplying the volume-adjusted expected cost by the probability of receiving a given procedure.

The volume-adjusted expected cost was calculated by using the procedure’s base price, adverse event rate, adverse event incremental cost, and mean procedure count per patient. Based on the total average expected diagnostic assessment cost per patient for each group, SPN and LCDx, we were able to estimate the expected cost of follow-up diagnostic assessment. For the CyPath® Lung arm, we multiplied the number of TP and FP by a weighted total average expected diagnostic assessment cost per patient. For the purpose of this analysis, we assumed that larger pulmonary nodules have a higher likelihood of malignancy than the smaller range of nodules.^5^ Data from Gierada et al were used to estimate the proportion of patients with larger nodules in this cohort, which was categorized as individuals with nodules greater or equal to 10 mm; this was equivalent to 33% of the patient population.19 Hence, 33% of the follow-up costs were allocated to expenses associated with the LCDx group as they were deemed to have a higher rate of malignancy.

In the CyPath® Lung test arm, we added the cost of the test to the expected diagnostic assessment cost per patient to calculate total expected cost within the 1-year time horizon. We then multiplied that result by the number of individuals who were estimated to be true positive, false positive, true negative, and false negative (TP, FP, TN, and FN, respectively). We calculated 2 cost-saving values, one that accounts for FN individuals, and one that does not. The reason for this disaggregation is that those who receive an inaccurate, negative test result will incur costs associated with follow-up as their nodules are expected to grow and require attention within the 1-year time frame. We anticipate the occurrence of false-negative results among patients. While disease progression is not factored into this analysis, we address the associated costs incurred from follow-up visits within the 1-year timeframe of this model by including the estimated costs linked to follow-up care for those patients. This involves multiplying the number of patients who received a false-negative test by the estimated costs linked to follow-up care. In the LDCT-only arm, we multiplied the number of patients in the cohort who received a positive result by the expected cost of diagnostic follow-up. Cost savings were then derived by calculating the difference between the 2 groups.^5^

Finally, we conducted a deterministic sensitivity analysis and generated a tornado diagram. We varied key input variables one at a time while keeping other variables constant. This approach allowed us to assess the impact of each variable on the overall model outcome. Specifically, we identified the most influential parameters, adjusted them incrementally to their low and high values, and recorded the corresponding changes in the model’s results. The key parameters we used for this analysis included the expected cost of follow-up for patients who are expected to have a lung cancer diagnosis, the cost of CyPath® Lung test, adverse event rates for 3 procedures (CT scan, bronchoscopy, and surgical biopsy), the prevalence of larger nodules within the given cohort, and CyPath® Lung’s sensitivity. To obtain our low and high ranges, we adjusted values by 20%. A tornado diagram was then created to visually represent these variations, displaying the extent to which each variable influences the outcome. The purpose of this analysis was to identify and quantify the variables that have the most effect on the model’s results, thereby providing insight into areas that might influence decision-making while utilizing this new diagnostic tool.

## RESULTS

Cohorts of 138 571 patients each were directed through the CyPath® Lung diagnostic arm and through the LDCT-only arm. Adverse event rates remained the same for both the SPN group and the LCDx group, with adverse event rates of 28% for CT-guided biopsy, 6.57% for bronchoscopy, and 11.47% for surgical biopsies. The mean procedure count per patient varied between the SPN and LCDx group, with an average count of 1.43 procedures in the SPN group, vs 1.18 in LCDx. The probability of receiving a given procedure for the SPN group varied from 0.62% to 61.8% (**Table 1**). Total average expected diagnostic assessment costs per patient in the SPN group and LCDx group were $514.95 and $3730.66, respectively, from a Medicare perspective and $1299.07 and $9048.67, respectively, from a private payer perspective.

Based on the total average expected diagnostic assessment cost per patient for each group, we were able to estimate the expected cost of follow-up diagnostic assessment. In the CyPath® Lung arm, the expected cost of follow-up is $8 139 945 for individuals who are true positive, and $24 817 774 for false positive. When added to the cost of the CyPath® Lung test, the total expected costs are $12 095 106 (TP), $36 876 610 (FP), $88 431 467 (TN), $868 206 (FN) for a grand total of $138 271 389 for expected costs from a Medicare perspective. For the LDCT-only diagnostic arm, total expected costs are estimated to be $516 960 408. This results in a total cost savings of $378 689 020 per cohort or $2733 per patient when CyPath® Lung is used as part of the diagnostic protocol. When accounting for the individuals who were FN in the CyPath® Lung arm, given the possibility of them receiving further diagnostic procedures and treatment in the given time horizon of the model, the savings decreased slightly to $376 902 203 per cohort or $2720 per patient.

Total expected costs and savings were greater when calculated from the private payer perspective. Total expected costs, including both the cost of the CyPath® Lung test and the expected cost of follow-up, were $358 677 100. For the LDCT arm, the total expected costs of follow-up were $1 253 879 411. Setting aside the impact attributable to FN, total cost savings were $895 202 311 for the modeled cohort or $6460 per patient. When accounting for patients expected to receive an FN test, savings slightly decreased to $890 829 889 for the cohort or $6429 per patient.

**Figure 2.**
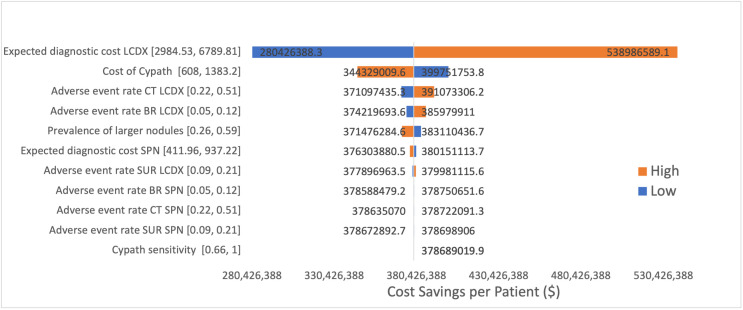
Deterministic Sensitivity Analysis (1-Year Results) Abbreviations: BR, bronchoscopy; CT, computed tomography; LCDX, lung cancer diagnosis; SPN, suspicious pulmonary nodules; SUR, surgical biopsy.

Based on a one-way sensitivity analysis of the model, we found that the most influential parameters on the outcome, specifically total cost offsets, were the expected cost of follow-up for patients diagnosed with lung cancer and the cost of CyPath® Lung itself, as illustrated below in the tornado diagram (**Figure 2**). These factors had the greatest impact on the variability of the model’s results, indicating their critical role in determining the overall cost-effectiveness of the intervention.

## DISCUSSION

The aim of this study focused on identifying cost savings associated with the introduction of a companion test to the current SOC pathway for individuals whose pulmonary nodules ranged from 6 to 29 mm. Clinical assessment of such nodules is associated with relatively high uncertainty, as rates of malignancy are lower in this group, but the benefits of earlier diagnosis are substantial.^28^ This model demonstrates that adding CyPath® Lung to the current SOC can help avoid potentially unnecessary follow-up diagnostics, resulting in substantial cost savings.

Economic models have some limitations, most of which stem from the accuracy of the input parameters used in the model. This model relied on 3 categories of input parameters: (1) test costs; (2) test probabilities; and (3) test complication probabilities. In terms of test costs, the model relied on direct measures of Medicare costs, but estimated private payer costs using an average Medicare differential. Although some private payers use similar multipliers, there is considerable variation in multipliers, and some private payers use alternative rate setting mechanisms. In the present analysis for private payers, we applied a multiplier of 2.64 that was retrieved from a recent literature review done by the Kaiser Family Foundation on how much more private insurers pay compared to Medicare. For the Medicare analysis, we used the 2024 Medicare stated reimbursement amount of $760 for the test’s CPT code (0406U).

Procedure probabilities were based on a study by Zhang et al where probability of receiving a given procedure was estimated for 2 groups, individuals with SPN at baseline and individuals with a confirmed primary lung cancer diagnosis. Limited biopsy data for this cohort may have impacted probability values. Our analysis relied on Zhang et al because of the rigorous methodology used in that study. They had a large sample size of over 30 000 patients and relied upon appropriate databases; however, we acknowledge that this study included some geographical constraints (ie, data were sourced only from the state of Louisiana). Insofar as healthcare utilization varies by state, it is possible that the Zhang et al study may not be broadly generalizable to other populations. Similarly, while the results are likely to be broadly generalizable to a national population, they may not be generalizable to specific subgroups based on sociodemographic characteristics. Insofar as lung cancer detection rates vary by sociodemographic factors, this is considered a limitation of the study.

Increased FP rates of lung cancer can result in unnecessary use of health resources and increases in procedure-related complications.^28^ The results of this study can aid in mitigating these risks, as it demonstrates the value associated with reducing FP results. Future studies should incorporate costs from data collected from private insurers rather than relying on a multiplier that is prone to fluctuations over time.

Ultimately, integrating companion tests to work in conjunction with the standard of care has the potential to improve outcomes by more efficiently triaging patients along the care pathway, resulting in possible reductions in delay of diagnosis, more favorable prognosis, and better patient outcomes.^29^ Moreover, it has implications for healthcare-related costs which differ across various stages of cancer diagnosis. For instance, an analysis evaluating average costs over 12 months across 11 cancer types (bladder, breast, colorectal, esophageal, kidney, liver, lung, ovary, pancreatic, prostate, and stomach) estimated $48 300 for first stage, $72 600 for regional stage, and $83 900 for distant stage.^29^

## CONCLUSION

This economic evaluation found that CyPath® Lung led to significant cost savings compared with the standard array of diagnostic testing. For high-risk individuals with pulmonary nodules ranging from 6 to 29 mm, the model shows that CyPath® Lung can result in a total savings of $2733 per patient in the Medicare population and $6460 per patient in the private commercially insured population. Incorporating this new diagnostic test has the potential to result in thousands of dollars per person and hundreds of millions of dollars overall in savings to health care associated with screening and diagnosis of lung cancer.

### Disclosure

The views expressed in this article do not necessarily reflect the official policy or position of the Defense Health Agency, the Department of Defense, the Veterans Health Administration, the Department of Veterans Affairs or any other government agencies.

## Supplementary Material

Online Supplementary Material
