# Supplementary material for: Economic Evaluation of a Novel Lung Cancer Diagnostic in a Population of Patients with a Positive Low-Dose Computed Tomography Result
Source: J Health Econ Outcomes Res. 2024 Sep 17;11(2):74–9. doi: 10.36469/001c.121512 (PMC11731590; doi:10.36469/001c.121512)
Supplement: Online Supplementary Material [file jheor_2024_11_2_121512_246175.pdf]

Journal of Health Economics and Outcomes Research

Online Supplementary Material

Economic Evaluation of a Novel Lung Cancer Diagnostic in a Population of Patients With a Positive Low-Dose Computed Tomography Result. *JHEOR*. 2024;11(2):74-79. doi:10.36469/jheor.2024.121512

Table S1: Cost Data Library: Medicare Cost and CPT Codes Associated with Each Procedure Type

Table S2: Cost Data Library: Private Payer Cost Associated with Each Procedure Type Using Multiplier of 2.64

Table S3: Incremental Complication Costs for Minor, Intermediate, and Major Complications

Table S4: Average Expected Diagnostic Assessment Costs per Patient with Suspicious Pulmonary Nodules from Private Payer Perspective

Table S5: Average Expected Diagnostic Assessment Costs per Patient with Lung Cancer Diagnosis from Private Payer Perspective

Table S6: Average Expected Diagnostic Assessment Costs per Patient with Suspicious Pulmonary Nodules from Medicare Perspective

Table S7: Average Expected Diagnostic Assessment Costs per Patient with Lung Cancer Diagnosis from Medicare Perspective

Table S8: Cost Impact Analysis from Medicare Perspective

Table S9: Cost Impact Analysis from Private Payer Perspective

This supplementary material has been provided by the authors to give readers additional information about their work.

This is an open-access article distributed under the terms of the Creative Commons Attribution 4.0 International License (CCBY-4.0). View this license's legal deed at http://creativecommons.org/licenses/by/4.0 and legal code at http://creativecommons.org/licenses/by/4.0/legalcode for more information.

Table S1. Cost Data Library: Medicare Cost and CPT Codes Associated with Each Procedure Type

Test/Procedure	OPPS National APC Payment, $	MD Professional Fee Only, $	2023 Cost/Payment, $	CPT Code
CyPath® Lung			760.00	0406U
CT scan	180.34	55.91	236.25	71260
PET/CT scan with FDG	1489.35	103.39	1592.74	78814
Fine needle biopsy	1499.55	84.04	1583.59	32400
Core needle biopsy	1499.55	151.48	1651.03	32408
CT-guided biopsy	1499.55		1617.31	32408
Bronchoscopy	3333.65	184.01	3517.66	31629
Mediastinoscopy	5168.00	319.00	5487.00	39402
Mediastinotomy	9724.03	789.23	10 513.26	39010
Thoracoscopy	5212.15	306.34	5518.49	32607
Thoracotomy	9724.03	800.42	10 524.45	32097
Surgical biopsy	7457.05		8010.80	32405

Abbreviations: CPT, current procedural terminology; CT, computed tomography; PET, positron emission tomography.

Table S2. Cost Data Library: Private Payer Cost Associated with Each Procedure Type Using Multiplier of 2.64

Test/Procedure	Total Private Payer Cost ($)	CPT Code
CyPath® Lung	2006.40	0406U
CT scan	623.70	71260
PET/CT scan with FDG	4204.83	78814
Fine needle biopsy	4180.68	32400
Core needle biopsy	4358.72	32408
CT-guided biopsy	4269.70	32408
Bronchoscopy	9286.62	31629
Mediastinoscopy	14 485.68	39402
Mediastinotomy	27 755.01	39010
Thoracoscopy	14 568.81	32609
Thoracotomy	27 784.55	32097
Surgical biopsy	21 148.51	32405

Abbreviations: CPT, current procedural terminology CT, computed tomography; PET, positron emission tomography.

Table S3. Incremental Complication Costs for Minor, Intermediate, and Major Complications

	Minor	Intermediate	Major
Cytology	9501.00	15 252.00	38 633.00
Bronchoscopy	7478.00	18 985.00	60 838.00
Surgical	10 634.00	24 841.00	63 034.00

Minor complications include incidence of pneumothorax, major complications includes hemorrhage.
Source: Huo J, Xu Y, Sheu T, Volk RJ,Shih YCT. Complication rates and downstream medical costs associated with invasive diagnostic procedures for lung abnormalities in the community setting. *JAMA Intern Med.* 2019;179(3):324-332.

Table S4. Average Expected Diagnostic Assessment Costs per Patient with Suspicious Pulmonary Nodules from Private Payer Perspective

Test/Procedure	Base Price, $	AE Rate, %	AE Cost, $	AE Expected Cost, $	Total Expected Cost (Base Price + AE), $	Mean Procedure Count Per Patient	Volume Adjusted Expected Cost, $	Probability of Receiving Procedure Type, %	Total Expected Cost, $
CT scan	624				623.70	1	623.70	61.80	385.45
PET/CT scan	4205				4204.83	1	4204.83	0.62	26.07
CT-guided biopsy	4270	28	2468.90	686.60	4956.30	1.43	7081.15	1.19	84.08
Bronchoscopy	9287	7	5444.87	357.55	9644.17	1.43	13 778.79	4.25	585.50
Surgical biopsy	21 149	11	2993.20	343.32	21 491.83	1.43	30 705.74	0.71	217.97
Total average expected diagnostic assessment cost per patient	1299.07								

Abbreviations: AE adverse event CT computed tomography; PET positron emission tomography.

Table S5. Average Expected Diagnostic Assessment Costs per Patient with Lung Cancer Diagnosis (from Private Payer Perspective

Test/Procedure	Base Price, $	AE Rate, %	AE Incremental Cost, $	AE Expected Cost, $	Total Expected Cost (Base Price + AE), $	Mean Procedure Count Per Patient	Volume Adjusted Expected Cost, $	Probability of Receiving Procedure Type, %	Total Expected Cost, $
CT scan	624				623.70	1	623.70	72.53	452.37
PET/CT scan	4205				4204.83	1	4204.83	1.34	56.34
CT guided biopsy	4270	28	2468.90	686.60	4956.30	1.18	5871.61	35.43	2080.58
Bronchoscopy	9287	7	5444.87	357.55	9644.17	1.18	11 425.23	40.06	4576.90
Surgical biopsy	21 149	11	2993.20	343.32	21 491.83	1.18	25 460.89	7.39	1882.47
Total average expected diagnostic assessment cost per patient	9048.67								

Abbreviations: AE, adverse event; CT, computed tomography; PET, positron emission tomography.

Table S6. Average Expected Diagnostic Assessment Costs per Patient with Suspicious Pulmonary Nodules from Medicare Perspective

Test/Procedure	Base Price	AE Rate	AE Cost	AE Expected Cost	Total Expected Cost (Base Price + AE), $	Mean Procedure Count Per Patient	Volume Adjusted Expected Cost, $	Probability of Receiving Procedure Type, %	Total Expected Cost, $
CT scan	$236				$236.25	1	$236.25	61.80	$146.00
PET/CT scan	$1593				$1592.74	1	$1592.74	0.62	$9.87
CT guided biopsy	$1617	28	$2468.90	$686.60	$2303.91	1.43	$3291.64	1.19	$39.08
Bronchoscopy	$3518	7	$5444.87	$357.55	$3875.21	1.43	$5536.57	4.25	$235.27
Surgical biopsy	$8011	11	$2993.20	$343.32	$8354.12	1.43	$11935.67	0.71	$84.73
Total average expected diagnostic assessment cost per patient	$514.95								

Abbreviations: AE, adverse event; CT, computed tomography; PET, positron emission tomography.

Table S7. Average Expected Diagnostic Assessment Costs per Patient with Lung Cancer Diagnosis from Medicare Perspective

Test/Procedure	Base Price, $	AE Rate, %	Adverse Event Incremental Cost, $	AE Expected Cost, $	Total Expected Cost (Base Price + AE), $	Mean Procedure Count Per Patient	Volume Adjusted Expected Cost, $	Probability of Receiving Procedure Type, %	Total Expected Cost, $
CT scan	236				236.25	1	236.25	72.53	171.35
PET/CT scan	1593				1592.74	1	1592.74	1.34	21.34
CT-guided biopsy	1617	28	2468.90	686.60	2303.91	1.18	2729.39	35.43	967.15
Bronchoscopy	3518	7	5444.87	357.55	3875.21	1.18	4590.87	40.06	1839.09
Surgical biopsy	8011	11	2993.20	343.32	8354.12	1.18	9896.94	7.39	731.74
Total average expected diagnostic assessment cost per patient	3730.66								

Abbreviations: AE, adverse event; CT, computed tomography; PET, positron emission tomography.

Table S8. Cost Impact Analysis from Medicare Perspective

Diagnostic Arm	Test/Procedure	True Positive	False Positive	True Negative	False Negative	Total
CyPath® Lung	Population, N	5204	15 867	116 357	1142	138 571
	Cost of CyPath® lung test	3 955 160.17	12 058 836.40	88 431 466.95	868 205.89	105 313 669.41
	Expected costs of follow-up diagnostic assessment, $	8 139 945.41	24 817 773.72	–	–	32 957 719.13
	Expected total costs, $	12 095 105.58	36 876 610.12	88 431 466.95	868 205.89	138 271 388.54
Tested positive						
LDCT only	Population, N	138 571				138 571
	Expected total costs of follow-up diagnostic assessment, $	516 960 408.48				516 960 408.48
Cost savings not accounting for false-negative individuals						
Cost savings (cohort), $	378 689 019.94					
Cost savings per patient, $	2 732.82					
Cost savings when accounting for false-negative individuals						
Cost savings (cohort), $	376 902 202.66					
Cost savings per patient, $	2719.93					

Abbreviation: LDCT, low-dose computed tomography.

Table S9. Cost Impact Analysis from Private Payer Perspective

Diagnostic Arm	Test/Procedure	True Positive	False Positive	True Negative	False Negative	Total
CyPath® Lung	Population, N	5204	15 867	116 357	1142	138 571
	Cost of CyPath® lung test, $	10 441 622.84	31 835 328.10	233 459 072.74	2 292 063.55	278 028 087.23
	Expected costs of follow-up diagnostic assessment, $	19 918 810.61	60 730 202.63	-	-	80 649 013.24
	Expected total costs, $	30 360 433.45	92 565 530.73	233 459 072.74	2 292 063.55	358 677 100.48
Tested positive						
LDCT only	Population, N	138 571				138 571
	Expected total costs of follow-up diagnostic assessment, $	1 246 214 693.26				1 246 214 693
Cost savings not accounting for false-negative individuals						
Cost savings (cohort), $	895 202 310.73					
Cost savings per patient, $	6 460.26					
Cost savings when accounting for false-negative individuals						
Cost savings (cohort), $	890 829 888.89					
Cost savings per patient, $	6 428.71					

Abbreviation: LDCT, low-dose computed tomography.
